# Development of a prototype for high-frequency mental health surveillance in Germany: data infrastructure and statistical methods

**DOI:** 10.3389/fpubh.2023.1208515

**Published:** 2023-07-14

**Authors:** Stephan Junker, Stefan Damerow, Lena Walther, Elvira Mauz

**Affiliations:** Department of Epidemiology and Health Monitoring, Robert Koch Institute, Berlin, Germany

**Keywords:** COVID-19, mental health, surveillance, automatic, smoothing, trends, prediction, spline

## Abstract

In the course of the COVID-19 pandemic and the implementation of associated non-pharmaceutical containment measures, the need for continuous monitoring of the mental health of populations became apparent. When the pandemic hit Germany, a nationwide Mental Health Surveillance (MHS) was in conceptual development at Germany’s governmental public health institute, the Robert Koch Institute. To meet the need for high-frequency reporting on population mental health we developed a prototype that provides monthly estimates of several mental health indicators with smoothing splines. We used data from the telephone surveys German Health Update (GEDA) and COVID-19 vaccination rate monitoring in Germany (COVIMO). This paper provides a description of the highly automated data pipeline that produces time series data for graphical representations, including details on data collection, data preparation, calculation of estimates, and output creation. Furthermore, statistical methods used in the weighting algorithm, model estimations for moving three-month predictions as well as smoothing techniques are described and discussed. Generalized additive modelling with smoothing splines best meets the desired criteria with regard to identifying general time trends. We show that the prototype is suitable for a population-based high-frequency mental health surveillance that is fast, flexible, and able to identify variation in the data over time. The automated and standardized data pipeline can also easily be applied to other health topics or other surveys and survey types. It is highly suitable as a data processing tool for the efficient continuous health surveillance required in fast-moving times of crisis such as the Covid-19 pandemic.

## 1. Introduction

The COVID-19 pandemic and non-pharmaceutical interventions to reduce transmission of the virus as well as the societal discourse on the pandemic had far-reaching impacts on populations worldwide. Questions about the potential consequences for mental health arose from the beginning of the outbreak (1–3). As the pandemic unfolded, it became clear that it was a long-term stressor with many phases and sometimes rapid changes in circumstances (4). Therefore, ongoing and high-frequency monitoring of mental health and other outcomes was called for, and several public health institutions began tracking mental health indicators at regular intervals, including in the US (5), France (6), and England (7). Importantly, this type of continuous, temporally finer-grained surveillance comes with particular requirements for data processing, estimate calculation, and output for interpretation.

A nationwide Mental Health Surveillance (MHS) was in conceptual development at the Robert Koch Institute when the pandemic began in Germany (8). In line with the established concept of public health surveillance (9), the aim of the MHS is to regularly and systematically quantify core mental health indicators in order to provide information on the development of population mental health as a foundation for public health action. While this surveillance system was not yet in operation at the start of the pandemic and not initially conceived for high-frequency updates on mental health, we implemented a strategy for mental health monitoring using monthly data from a series of population-based telephone surveys of adults in Germany to meet new information needs arising in the pandemic. The particular aim of this high-frequency surveillance approach is to provide information on possible changes in population mental health almost as they unfold in order to enable policymakers and health care practitioners to respond swiftly for optimal health promotion and prevention, particularly in times of crisis.

The first survey that we used for high-frequency monitoring was the third wave of the European Health Interview Survey conducted as part of the study “German Health Update” (GEDA 2019/2020-EHIS) for Germany, which began data collection almost exactly 1 year before the outbreak of the pandemic (10). The study continued until January 2021, which was beyond the official end of GEDA 2019/2020 - EHIS. Even though the survey was not designed for monthly reporting, adjustments to sample weighting allowed for the calculation of monthly representative time-varying estimates of various health indicators, including symptoms of depression, as shown in previous reports by researchers at Robert Koch Institute (11, 12). Subsequently, data for high-frequency mental health monitoring has come from the studies “COVID-19 vaccination rate monitoring in Germany (COVIMO)” (13) as well as GEDA 2021 and GEDA 2022 (14), all of which were designed for monthly reporting given pandemic-related monitoring needs.

Data on several mental health indicators from these surveys was used to build a prototype for mental health surveillance of the adult population in Germany on the basis of graphically represented, continuously updated time series of monthly estimates. The prototype was developed to meet the following criteria:

1. Output should be updated to include the most recent available data as fast as possible, requiring a highly automated data processing and estimate calculation pipeline.
2. The results should be generalizable to the adult population in Germany.
3. It should be possible to analyze developments over time adjusted for demographic changes with regard to sex, age, and level of education.
4. It should be possible to compare sociodemographic subgroups by sex, age, and level of education, standardized and unstandardized for the respective other two characteristics.
5. The results should be as temporally fine-grained as possible.
6. Although our prototype’s objective is to identify changes, it should not be overly responsive to minor and random fluctuations, as this would complicate the graphical interpretation. In other words, it necessitates the use of a technique for smoothing short-term fluctuations.
7. The results need to be in a format suitable for graphical presentation, for example via a dashboard.

In this paper, we describe the prototype comprising an automated data pipeline from data collection, data preparation, and calculation of estimates to output creation. Furthermore, the statistical methods used are described. These include a weighting algorithm, a linear and a logistic regression model used to make predictions on a standard population for a moving three-month window, and a generalized additive model (15) with smoothing splines employed to make predictions on a standard population for a weekly interval.

## 2. Methods

### 2.1. Data and software

**Figure 1:**
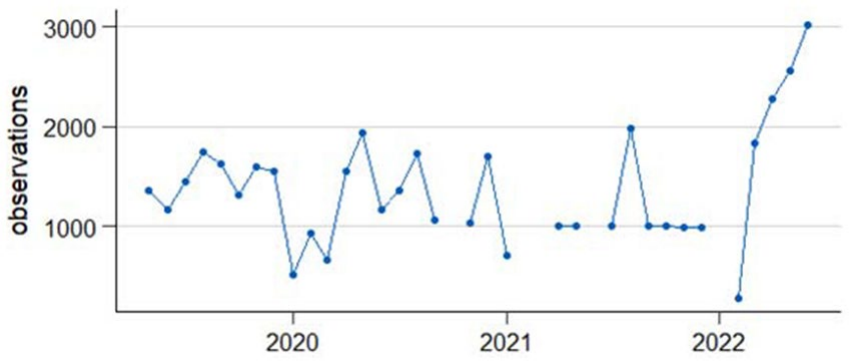
Observations per one-month period ranging from middle of the month to middle of the following month.

The data used for the analyses are from telephone health surveys conducted on behalf of the Federal Ministry of Health (BMG) as part of the nationwide health monitoring program German Health Update (GEDA) (10, 14, 16, 17) and COVID-19 vaccination rate monitoring in Germany (COVIMO) (18). The number of observations per monthly period ranging from the middle of one month to the middle of the following month between April 2019 and April 2022 are shown in Figure 1.

SAS SE^®^ software, version 17.1^1^ is used for data management and data cleaning procedures as well as to perform adjustment weighting. All other data processing and some of the analysis steps are carried out in R version 4.1.2. (19), RStudio 2022.02.1.461 (20). Several packages available for R are used. For data preparation and pipeline programming, dplyr 1.0.7 (21), rlang 0.4.12 (22), readstata13 0.10.0 (23), ISOweek 0.6–2 (24), and stringr 1.4.0 (25) are used. RStata (26) is used to transfer data back and forth between R and Stata. For analysis and prediction of the smoothing splines, mgcv 1.8.39 is used. The tool rmarkdown 2.11 (27–29) is used to structure analyses and graphs. We create the graphs with ggplot2 (30). Stata 17 (31) is used to estimate marginal predictions with confidence intervals on the basis of linear and logistic regression models.

### 2.2. Data pipeline

In order to meet the first criterion of promptly updating the output as soon as new data becomes available, we implemented a highly automated data pipeline. It consists of two parts, the data collection and quality assurance by the Epidemiological Data and Survey Center of Department 2 (EDC) of the Robert Koch Institute and external contractors as well as the data preparation and analysis for the purpose of mental health surveillance. In this section the work of the EDC is briefly summarized while the automated process for the MHS is laid out in more detail. At the end of the data pipeline estimation results are stored in a table format or as lists of tables, ensuring broad compatibility for further applications (criterion 7).

**Figure 2:**
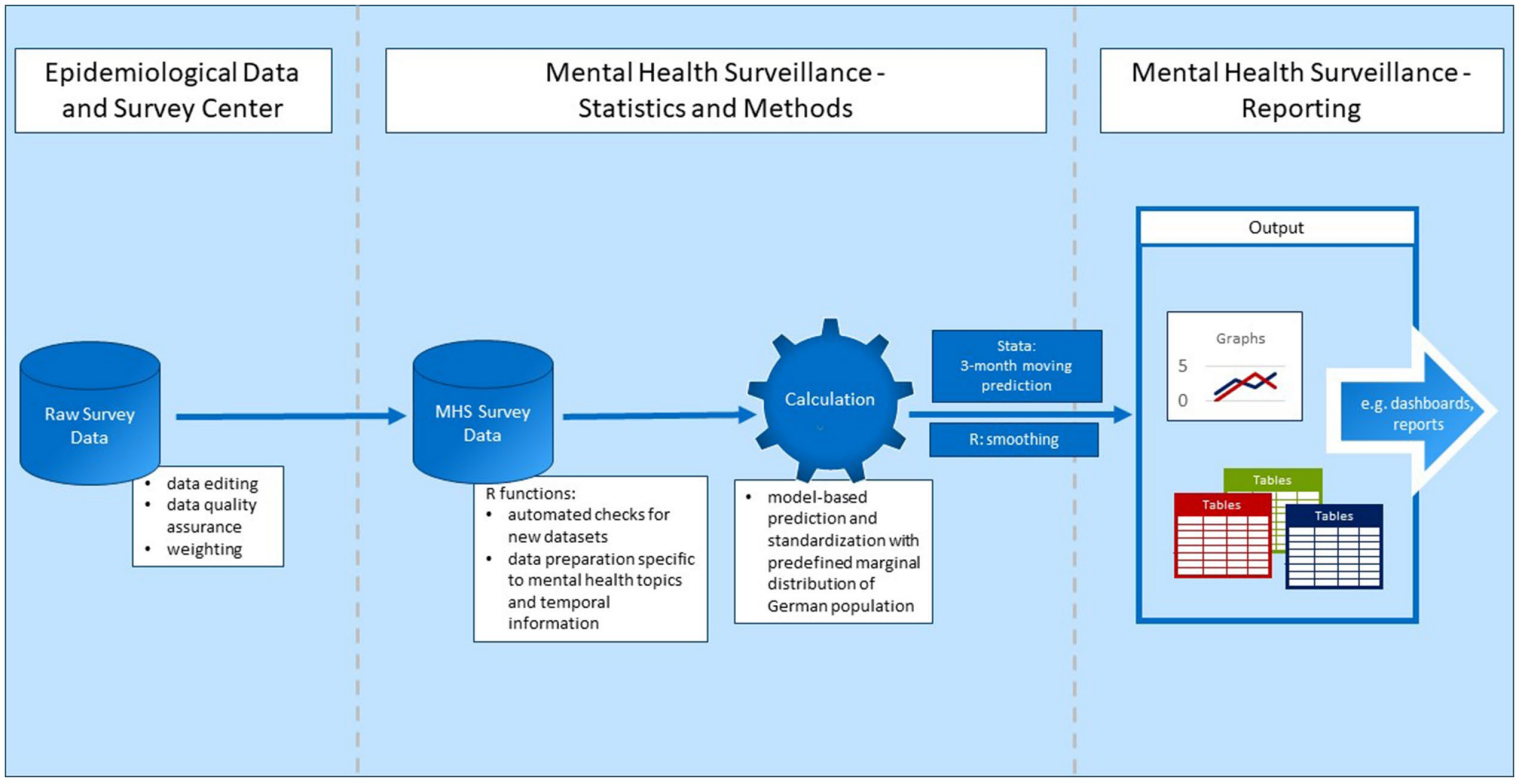
Data pipeline.

For the first part of the data pipeline, an external market and social research institute (USUMA GmbH) is contracted to conduct the telephone surveys, and provides the data to the EDC of the Robert Koch Institute. Figure 2 shows a simplified version of the data pipeline starting at the EDC.

Before the data is made available for analysis, the EDC performs standardized data editing and data quality assurance on the raw survey data. For example, implausible data is deleted or corrected, cases are cleaned, and new variables are generated (10). This only applies to data from the GEDA study, however. Because the COVIMO study was not integrated into the EDC’s data editing and data quality assurance procedures, these were performed by the authors. Because the study is conducted using computer-assisted telephone interviews which already include filtering of questions and value range checks, data inconsistencies are rare and data editing is limited to very few cases. Weighting factors are provided by the EDC for both data sources. The data is then made available in Stata and SAS format.

After the data has been made available by the EDC, it is prepared for the specific calculations performed within the MHS. Because the aim is to update the results as fast as possible on the basis of continuously collected data, data preparation needs to be mostly automatic and flexible. Thus, several functions and scripts were developed in R to perform the following tasks: First, an object (tibble) is created encompassing all necessary metadata for the survey data files. The metadata contains information on the location of the data and information for data processing, including an identifier specifying the function used for further processing of this data file. There are automatic checks for new data or data updates in predefined folders. When new data becomes available in one of these folders, a new row is automatically added. If neither updated data nor new data is found, the data processing stops. If, however, one of the two criteria are met, the data files are prepared separately from each other by the specified function, a new metadata file is created and saved, and a list of data frames containing the data from the different surveys and survey waves is created and later unpacked to a single data frame. Data is imported in the form of Stata files. Predefined value labels are omitted as special characters or extensive value labels are error-prone in R.

To prepare the MHS survey data, data editing is done in two stages. In the first instance of data editing, variables are coded into a standard format within individual data frames including data from only one data file, as mentioned above. Individual data frames are used because different data sources require different forms of preparation. For example, some variables are already generated in one data file but not in the other, so this has to be done retroactively only for these data files. Also, time identifiers for the interview date differ in format between the data files. The newly created time identifiers include a standardized string variable identifying the calendar week of the interview following ISO-norm 8601 (32) and the interview month, here operationalized as middle of one month to middle of the following month (see section 2.3.2).

The second stage of data preparation begins after all data files are combined into one: this stage of data preparation includes the naming of the different levels of categorical variables and, most importantly, the creation of additional time variables. Weekly and monthly identifiers are further processed to include information on the whole time series from start to finish, including all data gaps, as date, ordered factor, and numeric variables counting the weeks and months from the start of the time series.

The next step of the data pipeline covers the calculation of adjusted predictions for each three-month period (statistical details section 2.3.2) and a smoothed curve on a weekly basis (statistical details section 2.3.3). As Stata’s margin function is commonly used to estimate marginal predictions with confidence intervals, the calculation of the adjusted predictions is performed in Stata 17.1. For this purpose, every data frame contained in the abovementioned list is used to perform Stata calculations. We use the package Rstata (26) to facilitate communication and translation between R and Stata. This package enables initiation of Stata and the transfer of data frames from R to Stata. The created Stata files are then read back into R using the package readStata13. The calculation is performed with Stata do-files for linear and logistic regressions, including the calculations of margins, confidence intervals, and finally, the saving as a temporary data file. The required definitions of control variables, dependent variables, weights, and data used for predictions are managed with Stata macros which are specified in another do-file created with R. The Stata results are stored as a data frame including the predicted means and proportions (with confidence intervals) of the respective mental health indicator and the related time-identifying variables and stratification variables/levels. The estimates for the smoothed curves, on the other hand, are calculated in R because the smoothing algorithm used is included in the R package mgcv (33) and not implemented in Stata. The data frame including all data from the individual data files is used for this calculation.

The final step in the data pipeline is to save the output in the required formats. Because our major use case for further processing is the publication of the result on a dashboard, the results will eventually be saved in an SQL database with stratification by time as monthly and weekly smoothed data and the grouping parameters. At present, the dashboard is still in development, and the data is saved as an RData file. Plotting functions were programmed as add-ons to create publication-ready graphs in an R-Markdown file (27–29) using ggplot2 (30). They show the time series of three-month moving average predictions and the weekly smoothed curve for every mental health indicator stratified by sex, age group, or education standardized and unstandardized.

### 2.3. Statistical methods

This section details methods used for data weighting and output calculation within the semi-automated data pipeline described above. In what follows, the rationale behind these methods is briefly outlined. Subsequent sections describe different methodological steps and decisions in greater depth.

Criteria 2–5 specified above require that the prototype produce a temporally fine-grained output, representative estimates which can be compared over time, and standardized as well as unstandardized estimates for comparisons between the subgroups of interest. With the estimation of monthly weights by the EDC detailed in 2.3.1 below, fluctuations in participation between groups defined by regions, sex, level of education, and age over time can be addressed. Weighting also ensures representativity by approximating the German population and correcting for different probabilities by design.

The criterion of maximum temporal resolution and limitations of our dataset informed our first decision about the output calculation (see section 2.3.2): While producing a new estimate monthly would be desirable, our data includes too few observations in one-month periods for a direct estimation of monthly means and proportions: with a small monthly n, cell counts can simply be too low, and time series risk becoming noisy with random fluctuations. Data gaps and months with a particularly small number of observations pose added challenges. To base calculations on larger subsamples and thereby reduce the risk of random fluctuations, we opted to calculate estimates for moving three-month windows. This technique provides more observations for estimation, smooths time series by reducing random fluctuations, and still produces new estimates for every month.

Standardization between subgroups (criterion 4) as well as ensuring representativity and comparability (criteria 2 & 3) over time given the specific time windows used in analyses necessitates the use of regression modelling (see section 2.3.2) rather than a straightforward calculation of means and proportions. For standardization between the subgroups defined by one characteristic – for example sex – by the remaining two, we used predictions on a standard population (see section 2.3.4). Regression models including the sociodemographic characteristics as independent variables provided the foundation for standardization (34, 35). This procedure also ensures standardization across time when the official population distributions are changed within the EDC’s weighting process. It acts as a secondary safeguard to address changes in distribution between the monthly samples, particularly when weights cannot be estimated for the exact time periods we used.

Sample means are sensitive to outliers and can be very sensitive to short-term fluctuations depending on sample size. For better separation of signal from noise (criterion 5) we considered several additional, less sensitive smoothing techniques and chose a thin plate smoothing spline because we found this method to strike a good balance between best fit and smoothing (see section 2.3.3). These choices and the methods are explained in more detail in the following sections.

#### 2.3.1. Data weighting

Data weighting is necessary to meet criterion 2 (“The results should be generalizable to the adult population in Germany”). The EDC’s data weighting procedure considers two aspects of sampling bias: (1) different selection probabilities of participants (design weighting) and (2) different likelihoods of participaton within different population subgroups (adjustment weighting).

Despite random sampling in the sense that random phone numbers were dialed, study participants have different probabilities of having been selected into the samples at hand (selection probability). The telephone studies used here recruit participants via a dual-frame approach, using landline phone numbers and mobile phone numbers. The probability of drawing a specific phone number is a major part of the overall selection probability of each respective individual survey participant. In the mobile phone frame, the individual selection probability also depends on how many mobile phone numbers each participant has. In addition, the number of persons using the phone number called for recruitment plays into selection probability. The selection probability in the landline frame is also dependent on the probability of selecting a specific individual from each contacted household. This, in turn, is determined by household composition and household size. Design weighting compensates for the different selection probabilities of participants, in that persons with a lower selection probability represent more people from the population than persons with a higher selection probability. Due to data policy and data privacy reasons, information on sampling frame and household composition are not available to the EDC. The contracted market and social research institute therefore provides design weights calculated as described in Häder et al. (36). The weights are calculated independently for the GEDA and COVIMO studies.

Secondly, the probability of participation is not the same across participants because willingness to participate in a survey and reachability may differ according to characteristics such as region, age, sex, or level of education. With regard to the aim of assessing time trends, it is additionally important to note that the demographics of the sample might change over time or differ between time periods because participation probabilities for individuals from specific groups might vary with time, for example, due to external influences such as lockdowns and working from home. The different levels of participation may lead to biased results if these characteristics are associated with the target outcome. In adjustment weighting, the differences in willingness to participate are considered by matching the sample to the population distribution of selected characteristics. In more general terms, this means that the sample is calibrated on the basis of nonresponse in order to increase the precision of estimators. This requires variables that are captured in the survey and whose true population values are known. The population distributions are based on statistics from the Federal Statistical Office (Destatis) and the German Microcensus (37). The adjustment is carried out for each month in the GEDA19/20 survey and for each survey wave of the other surveys which approximately cover the period of one month. The adjustment weighting is performed in several iterative steps according to the so-called “raking” procedure (38), which are carried out repeatedly one after the other. The adjustment levels are described in Table 1.

**Table 1:** Adjustment levels.

Level	Characteristics
1	Sex × age group (18–29, 30–39, 40–49, 50–59, 60–69, 70–79, and 80+ years of age)
2	Age group (18–29, 30–59, and 60+ years of age) × ISCED^*^ (lower, middle with/without A-levels, high)
3	Nielsen Areas of Germany (Northwest, North Rhine-Westphalia, Center, East (North), East (South), Bavaria, Baden Wuerttemberg) × municipality size (rural, small-town, medium-town, metropolitan)
4	Federal state of Germany (combined: Schleswig-Holstein & Hamburg, Lower-Saxony & Bremen, Saarland & Rhineland-Palatinate, Brandenburg & Mecklenburg-Western Pomerania, Saxony-Anhalt & Saxony & Thuringia) × age group (18–39, 40–59, and 60+ years of age)

^*^UNESCO Institute for Statistics (2012) International Standard Classification of Education: ISCED 2011. UIS, Montreal, S. 8.

It is possible that the number of observations in an adjustment level is small or zero. The calculation cannot handle empty adjustment cells because there must always be at least one observation to which the distribution can be fitted. In addition, an insufficient number of observations can lead to extreme values for the weighting factors. If the number of observations in an adjustment level for a time point is less than ten, a check is run as to whether the weighting factor values are zero or greater than a factor of ten. A weighting factor of zero cannot be used in analyses, since corresponding participants are thus practically excluded. On the other hand, participants with large weighting factors have excessive impact on the analyses. Another problem with a small number of observations in an adjustment cell is an endless iteration process in the raking procedure, which is here limited to 300 iterations. To address these problems arising from insufficient or zero observations in an adjustment cell, extreme values in the weighting factor, or an exceeded iteration limit, age groups are combined for the survey time point and adjustment level in question in order to increase the number of observations.

Missing values in any of the variables relevant for weighting are not permissible in the weighting procedure. Therefore, these must be assigned to a category or imputed. For education (ISCED), missing data are assigned to the middle category. Missing values in the self-declaration of federal state are imputed automatically, according to the distribution of federal states in Germany. Missing values in the political municipality size category are imputed based on the respective distribution in the federal state.

The adjustment weighting is performed with SAS SE software, version 17.1, using predefined syntax and macros for the raking procedure and distributional checks. It is performed after the data editing and quality assurance by the EDC.

#### 2.3.2. Linear and logistic regressions for three-month windows

The time series that this prototype outputs encompass monthly estimates; specifically, monthly predicted mean values or proportions based on Stata’s margins function for three-month windows. Periods of data collection within the surveys used for the prototype happen to begin roughly in the middle of the respective months. To optimize the number of cases per month, a monthly period is therefore defined as the middle of a month to the middle of the following month.

Linear and/or logistic regression models are fitted for each mental health indicator as the basis for prediction. In order to avoid bias due to changes in sociodemographic factors sex, age, and level of education between the monthly time periods and for the prediction of stratified values, the regression models include a list of covariates. For each individual *i* the linear regression model for metric mental health indicators is defined as:


(1) $$Y_{i}=\beta_{0}+\beta_{1}x_{1i}+\cdots +\beta_{j}x_{ji}=B^{T}X_{i}^{a}$$

and the logistic regression model for binary mental health indicators is defined as:


(2) $$\mathrm{logit}\left(Y_{i}\right)=\beta_{0}+\beta_{1}x_{1i}+\cdots +\beta_{j}x_{ji}=B^{T}X_{i}^{b}$$

where $B=\left(\beta_{0},\;\beta_{1},\ldots ,\;\beta_{j}\right)\;$is a vector of regression parameters and $X_{i}^{a}\;$and $X_{i}^{b}\;$ are vectors of auxiliary covariates. The latter includes age groups, sex, and level of education as well as their interactions. The covariates of interaction differ between the linear and logistic regression models: the linear regression (1) includes all combinations of age group, sex, and education ($X_{i}^{a}$), while the logistic regression (2) contains only two-way interactions ($X_{i}^{b}$) because otherwise, cells with zero observations are very likely to occur and prevent marginal prediction.

Some of the mental health indicators show a small prevalence of cases as defined by cutoffs, particularly within certain subgroups. For example, in one three-month period (centered on May/June 2021), 4% of men screened positive for possible anxiety disorder. Given the relatively small numbers of cases per survey month, this necessitated estimation for a three-month period rather than a one-month period in order to minimize random fluctuations (criterion 6). Following the procedure of centered moving averages (39), the observations from the previous and following month are defined as the period for the corresponding month. As the moving three-month windows overlap, regressions are separately fitted for each three-month window based exclusively on observations within it. Thus, for a survey period with M months, t *= 1 …*M separate regressions model estimates will be derived. At the beginning and at the end of a survey or due to interruptions during the survey, it is not always possible to sum up three months for each estimate in the time series. In these cases, the estimation is calculated based on two months if observations of at least two of the three months are available. The model results are then used for average predictions on a standard population as described below.

#### 2.3.3. Smoothing

The method of moving predictions is an intuitive way to smooth a time series (criterion 6). However, this smoothing technique is still very sensitive to outliers and might lead to overfitting the data and noisy time series from which the actual trajectory is difficult to detect (40). To prevent overfitting and thus misinterpretation due to random fluctuations, another smoothing technique is applied to our time series. Another disadvantage associated with the moving three-month windows is their limited utilization the available temporal information. The temporal information used is whether an observation falls within a given three-month window or not, disregarding its placement within that timeframe.

While sample size restricts the temporal resolution of the estimates, the data contains information down to the level of weeks. When time is not segmented into periods, but treated as a continuous variable, this information can be used despite small sample size at that level. In light of continuous data collection and the requirement of automatic output creation as well as its function in facilitating the interpretability of the time series output, the following criteria for smoothing were determined:

1. The smoothing technique needs to work automatically without manual specifications.
2. To ensure the accuracy of interpretation the smoothed curve should not introduce bias by either being overfitted to the data or oversmoothing and thus failing to capture important developments. Ideally, there is an objective criterion for optimization.
3. The smoothed curve has to be as stable as possible for time points in the past when new data is added but still be locally adaptive.

Several candidate smoothing techniques were considered on the available data: polynomials, restricted cubic regression splines, and smoothing splines. Polynomials did not fulfill the criteria because the curve is not smoothed locally. A polynomial function defines the shape of the curve globally. For example, a quadratic function will always produce an (inverted) “U”-shaped curve. Thus, an adaption of the curvature at some regions of the curve always necessitates a change in the whole curve. Also, this technique requires a decision about the ideal degrees of the polynomials with every new time point as well as checks for fit to data to prevent substantial changes in the curve for the previous point in the time series when new time points are added. A way to automate the determination of the ideal degree of the polynomial is to treat it as a tuning parameter and use cross validation: the model is fitted to one part of the data, and then the prediction is evaluated on another part of the data, for example using the root mean square error. This process is iterated for a predefined choice of values. However, this process is computationally time consuming and might still lead to substantial changes for the existing time series when a time point is added. Restricted cubic regression splines are locally adaptive as the function is fitted to different parts of the curve marked by knots. The regions before the first and after the last knot are restricted to linearity as the splines often show erratic behavior in these regions. The knots between which the function is fitted need to be defined and potentially manually adapted when new data points are added (41, 42). The necessity to make choices regarding the number of knots and their placement poses a disadvantage to a process that aims to be highly automated. One possibility, however, is to use a fixed number of knots with a default placement. Stone (43) showed that five knots should be sufficient for most scenarios a recommendation for default placement of knots is provided by Harrell (44).

As another method, we considered smoothing splines. Unlike cubic regressions they do not require knots; instead, a smoothing parameter controls the smoothness. We chose a smoothing spline with the basis function of a thin plate spline over other smoothing spline approaches because it is both theoretically well-founded and particularly suited to our needs given that the approximations developed by Simon Wood (42) made thin plate regressions computationally efficient so that they can also be used for large data sets. This technique has a very good level of accuracy, though the curves produced are not as smooth as other automatic smoothers (45). The smoothing parameter can be estimated automatically and simultaneously with the whole model by either using restricted maximum likelihood (REML) or generalized cross validation (46). This is a major advantage because it enables automatic estimation and avoids any manual presetting such as defining knots or degrees of polynomials. Thus, over-and underfitting to the data can be avoided without defining different choices of parameters for cross-validation. As generalized cross validation is prone to undersmoothing, we chose REML (46–48).

**Figure 3:**
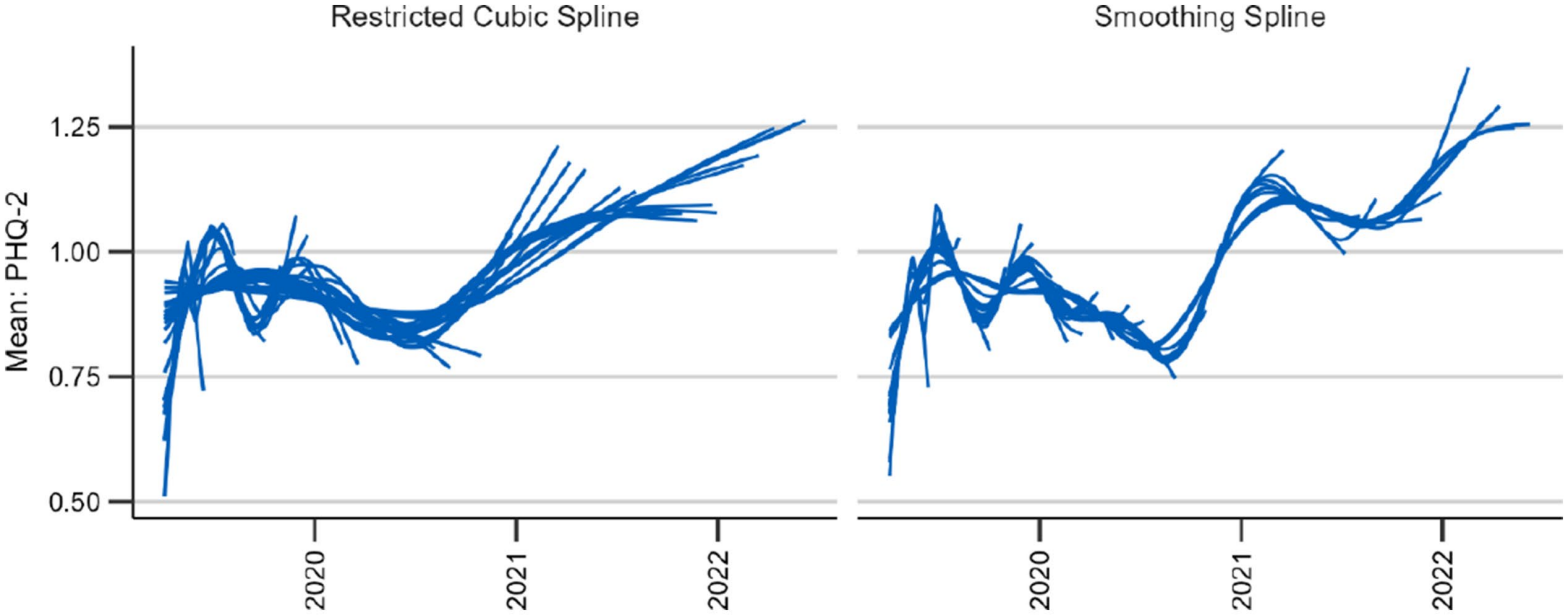
Behavior of two candidate splines with 39 updates every 4 weeks beginning after 12 weeks.

In order to determine the more suitable spline to use between restricted cubic splines and thin plate smoothing splines, we examined their behavior on recent time periods when new data was added. As mentioned in the criteria, data points added to the time series should only have a limited effect on preceding estimates. Also, the longer the calculations date back, the less pronounced the changes should be. We expected the smoothing splines to better meet this criterion because it is the ideal smoothing parameter that changes with new data, not the placement of knots, which we expected to have a higher impact. Moreover, the restricted cubic splines are restricted to linearity before and after the first and last knot, making abrupt changes more likely. We tested this hypothesis with data for a brief depression screening instrument, the Patient Health Questionnaire-2 (PHQ-2) (49). We simulated the addition of new data every week starting with a time series spanning 10 weeks (see Figure 3). For both the smoothing and the cubic spline, we see that new data changes the course of the curve. However, for the restricted cubic splines, it takes more updates for the estimates to change to fit to the later course of the curve. This is the case, for example, at the end of 2020 and the beginning of 2021 in the time series. The trend adapts with the addition of a single week of new data using smoothing splines, whereas this takes several weeks using the cubic spline (Figure 3).

**Figure 4:**
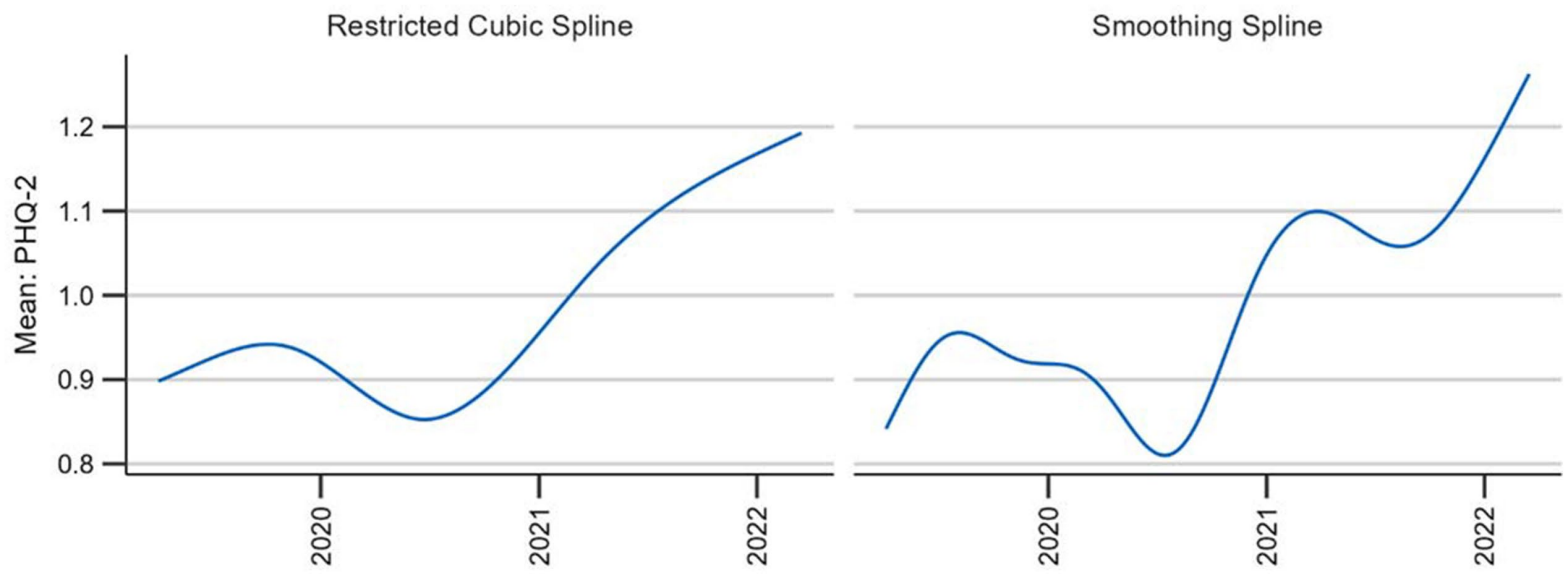
Comparison of two candidate splines across the entire observation period.

To examine the overall behavior of the two techniques we also plotted the curve with the two different splines for the entire observation period (see Figure 4). Although the general trends remain consistent, the smoothing spline provides a more detailed view. This in itself is neither an advantage nor a disadvantage; however, as the smoothing spline technique provides a mechanism against under-and overfitting, it seems that the restricted cubic spline underfits or oversmooths the data. Thus, the smoothing splines meet our criteria best.

To allow for separate smooths for different sociodemographic groups, we used factor-by-curve interactions (50) or varying-coefficient models (51). The thin-plate splines were then used as part of a general additive model (GAM) (15) specified as follows in pseudo-code:


$$\begin{array}{c} Y=s\left(week,\;\;by=interaction\left(agegroup,\;\;sex,\;\;education\right)\right)\\ +\;\;agegroup*sex*education \end{array}$$

#### 2.3.4. Prediction and standardization

In order to obtain standardized outcomes for the mental health indicators, the results are not estimated with the actual survey data, but with a standard population. This allows for direct standardization between time periods and between subgroups defined by sex, age, or level of education. For example, estimates for different age groups are adjusted for differences in sex and level of education (criteria 3 and 4). The estimates from the linear and logistic regression models as well as the general additive models for Gaussian and binomial distributions based on the survey data described above are used for these predictions on a standard population. The standard population is derived based on the latest available German microcensus data containing the population distribution by age group, sex, and education. The time series presented below were estimated using 2018 microncensus data (52).

This standard population is the basis for weekly and three-month predictions. The predictions are then averaged over the whole population as well as within each population subgroup by sex, age group, and level of education. Therefore, this method ensures standardization for age, sex and level of education over time. Consequently, older predications will undergo changes when the standard population is updated.

For direct standardization between the subgroups, predictive margins (35, 53) are calculated. The only difference to the procedure described above is that the standard population is used for each subgroup, treating all the rows as belonging to the subgroup in question. For example, predictions for the male population treat all individuals in the standard population as if they were male and vice versa for the female population (54). The Stata command “margins” is used for both the prediction with and without standardization between subgroups because the estimation of confidence intervals is already integrated into this command. With low or high prevalence, the confidence intervals estimated by the delta method may produce lower limits beyond zero and upper limits exceeding one. This is why resulting confidence intervals are constructed by means of a logit transform (55) using the undocumented command “coef_table, citype (logit).”

For the predictions of weekly values for the smoothed curve we wrote an R function using the following formula from Graubard and Korn (53):


$$PM\left(r\right)=\frac{\sum_{i=1}^{S}w_{i}g\left(r,\;Z_{i},\hat{\theta }\right)}{\sum_{i=1}^{S}w_{i}},$$

where g(r, Z, $\hat{\theta }$) is the predicted value which depends on $\hat{\theta }$ the survey weighted estimator for the model parameters, Z_i_ the S distinct values of the covariates for which the distribution in the external distribution is known, and r the value of the subgroup for which the mean or proportion is to be be predicted. When the prediction is standardized between subgroups, the weighting factor $w_{i}$ represents the number of individuals in the standard population with a distinct value of Z_i_. In case of an unstandardized prediction, the weighting factor only includes weights with distinct values of the covariates in subgroup r. For prediction of the whole population without stratification r can be removed from the formula.

As the calculation of confidence intervals of predictive margins with GAMs and survey design is beyond the scope of our project, only the point estimates are calculated.

#### 2.3.5. Missings

Missings are handled differently depending on the type of variable. While only interviews with information on sex and age are included in the sample, level of education is imputed by assigning missing values to the middle category, in accordance with the weighting procedure described above. Observations with missing values in the mental health indicator are excluded from analysis. If patterns of missingness in these variables are dependent on age, sex, education, or their interactions, exclusion should not result in bias because the estimation controls for these factors.

## 3. Results

With the prototype described above, it is possible to provide automatic updates of time series with each survey wave and produce graphical representations of the development of mental health indicators for Germany’s population [please see (56) for results]. To generate monthly estimates, data from moving three-month windows is used for the calculation of a linear or logistic regression model adjusting for age group, sex, and level of education.

This three-month window moves along one month at a time for the calculation of the next three-month estimate until the last month with observations enters the window. If only one month is missing in the three-month window, calculations are still performed; if two months are missing, no calculations are performed and there is a gap in the time series. The visual output of these three-month average predictions is intuitively understandable to non-experts and has the benefit of providing discrete estimates for specific intervals. They also include confidence intervals, which provide important information for visual inspection.

**Figure 5:**
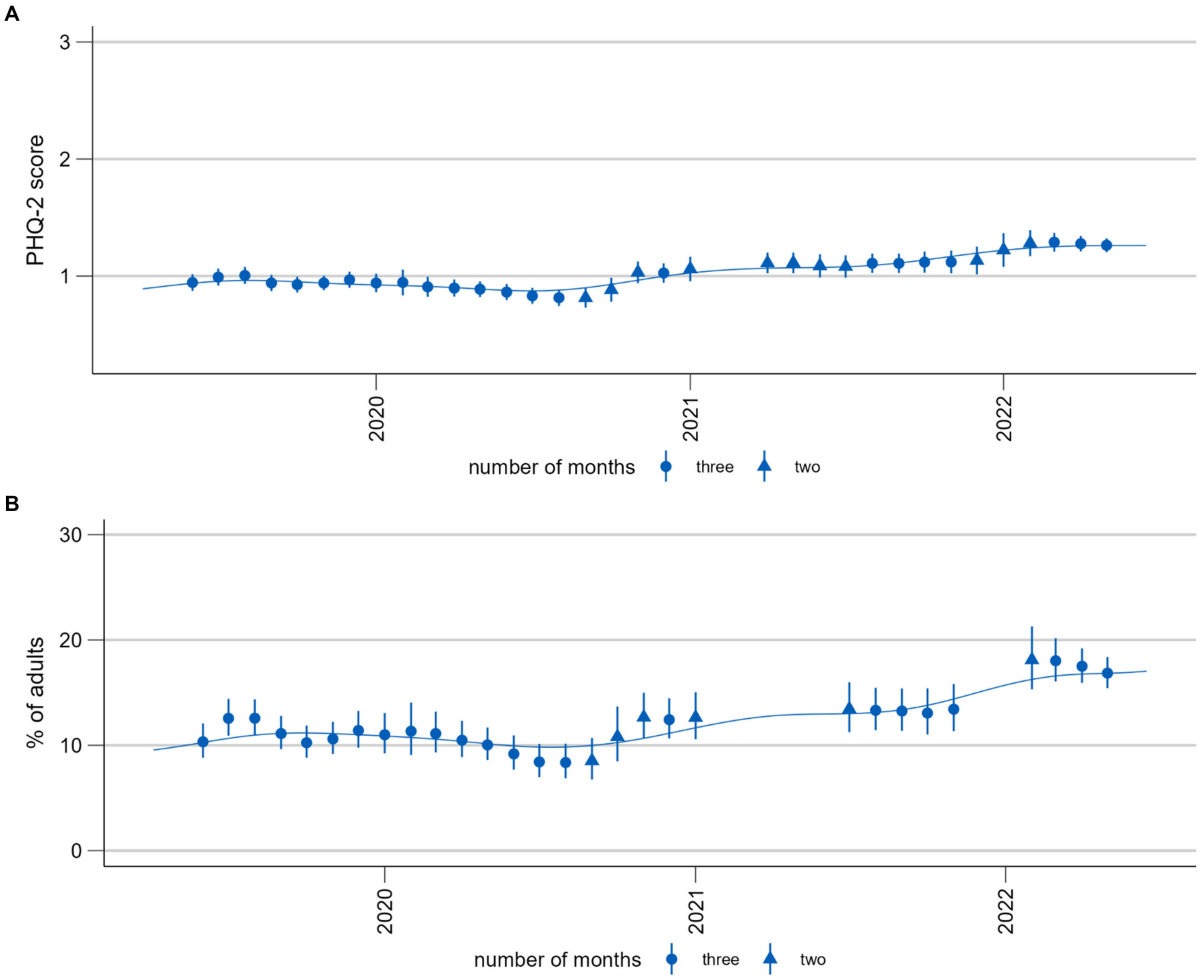
Time trends of symptoms of depression (PHQ-2). **(A)** Population mean (range 0–6). **(B)** Proportion of population screening positive for possible depression (PHQ-2 > 2). Please note that these time series are presented here for the purposes of illustrating and discussing a methodological approach. Please see another study for the results of these analyses (56).

**Figure 6:**
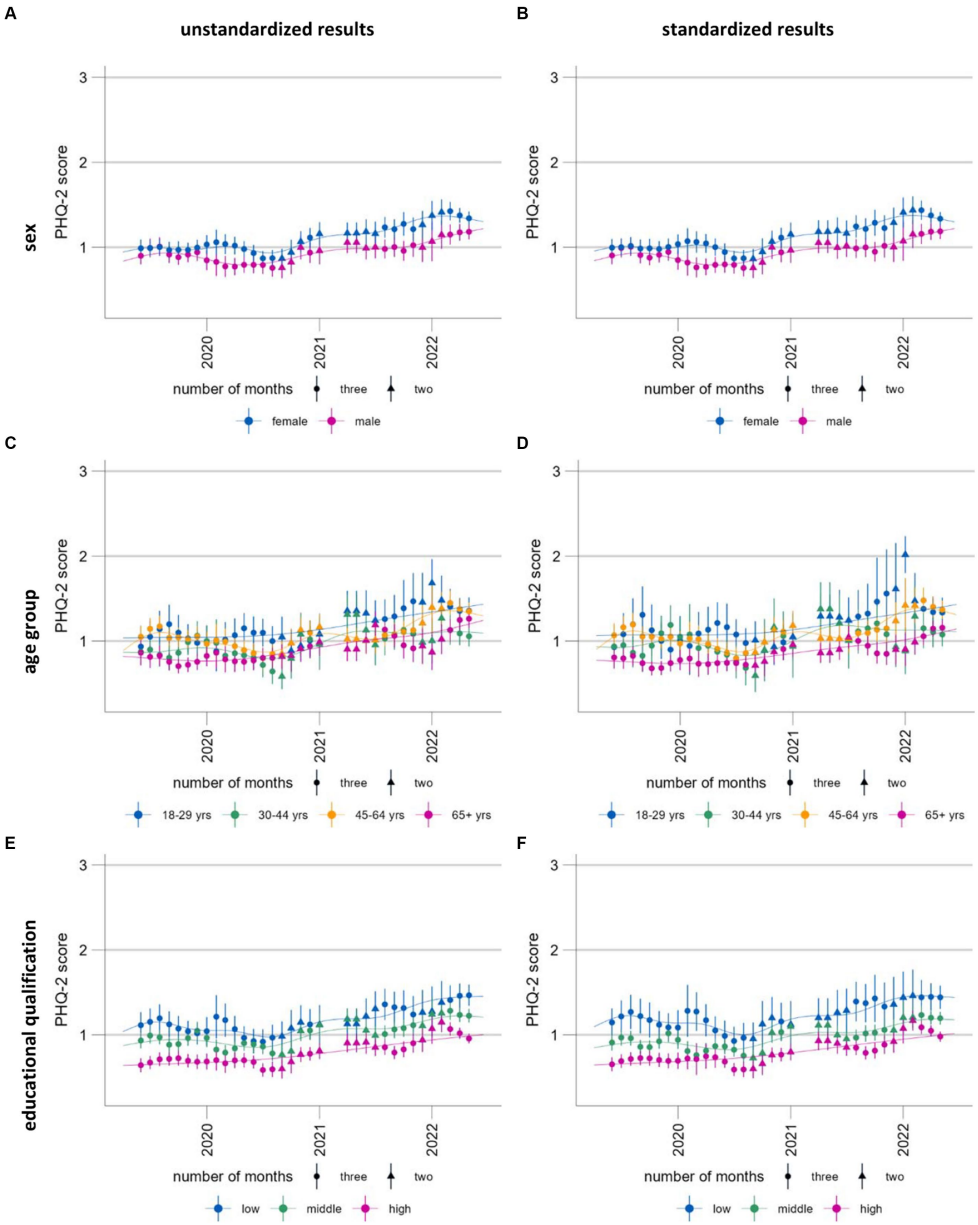
Time trends of symptoms of depression (population mean PHQ-2, range 0–6) stratified and standardized or unstandardized by sex **(A, B)**, age group **(C, D)** and educational qualification **(E, F)**, standardized **(A, C, E)** and unstandardized results **(B, D, F)**. Please note that these time series are presented here for the purposes of illustrating and discussing a methodological approach. Please see another study for the results of these analyses (56).

Figure 5 shows the predicted three-month averages and proportions for the time series monitoring depressive symptoms measured using the PHQ-2. Comparing these two time series, the most apparent difference is that there are fewer estimates for the proportions. This is due to empty cells which are more likely to occur with a dichotomous predictor. Using mean values therefore has the advantage of producing time series with fewer interruptions. By contrast, proportions have the advantage of being easier to interpret because they are based on a validated cutoff point indicating potential clinical significance (57). Although the technique of a moving average results in some smoothing, further aids in the visual differentiation between signal and noise would be beneficial, particularly with regard to stratified results (see Figure 6).

**Figure 7:**
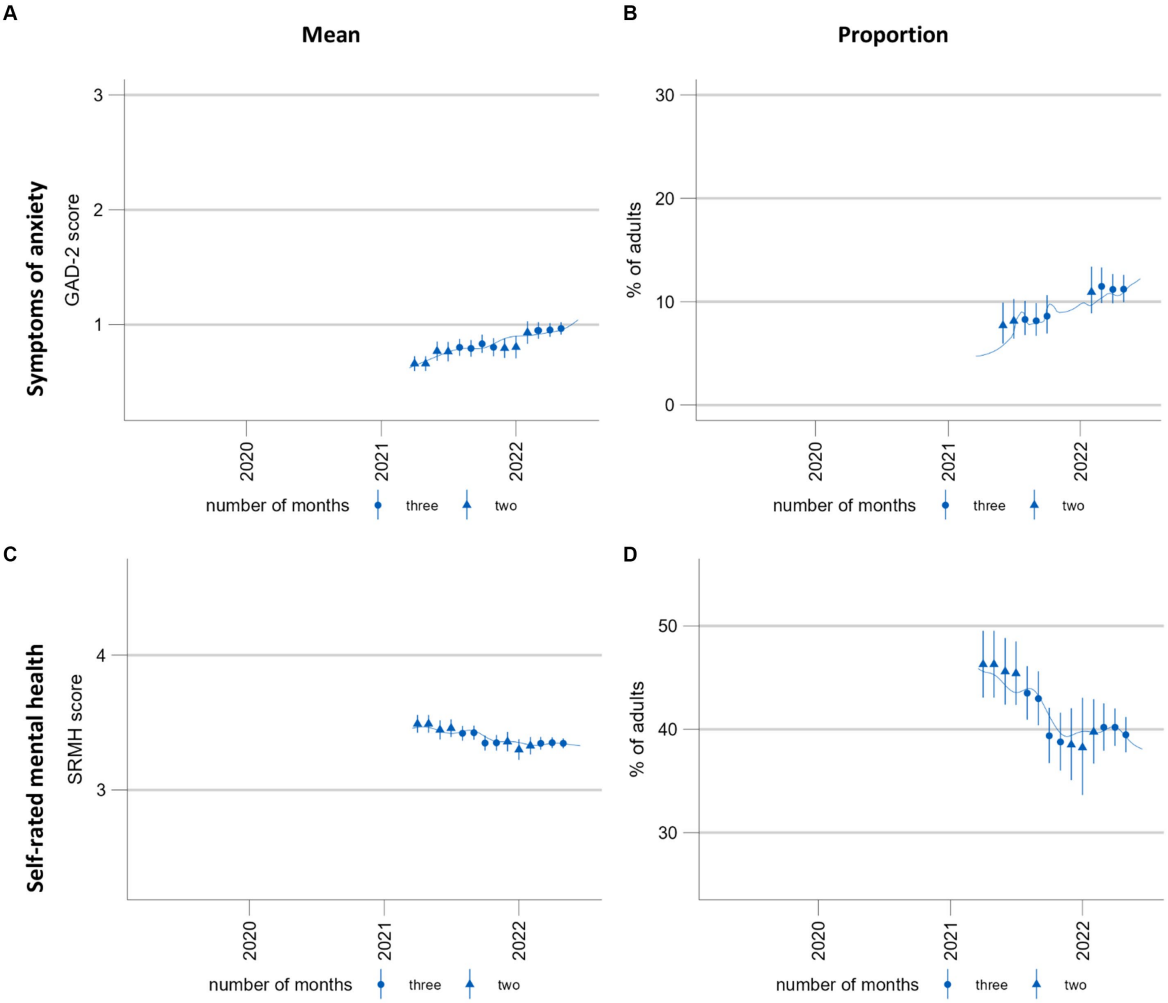
Time trends of symptoms of anxiety and self-rated mental health. **(A**,**C)** population mean GAD-2 scores (range 0–6) and mean SRMH scores (range 1–5), respectively. **(B**,**D)** show the proportion of the population screening positive for possible anxiety disorder at a GAD-2 score of >2 and those rating their own mental health “very good” or “excellent,” respectively. These time series are presented here for the purposes of illustrating and discussing a methodological approach. Please see another study for the results of these analyses (56).

This illustrates the need for a technique which provides more smoothing without overfitting. The addition of smoothing splines facilitates the differentiation between signal and noise to identify trends. An additional benefit is that all available temporal information is used to estimate the curve, providing information at a higher temporal resolution. However, with shorter time series such as the self-rated mental health time series [Figures 7C,D, measured using a single item (58)], this additional temporal information smooths the time series less effectively than the moving three-month averaged predictions: the smoothing curves reflect fluctuations between monthly estimates, which the moving averages, by definition, are unable to show at the same temporal resolution. For shorter time series with low cell counts such as the time series for the screening instrument Generalized Anxiety Disorder-2 [GAD-2, (59)] (Figures 7A,B), the general additive model fails to provide interpretable results for proportions; however, continued data collection may resolve this problem. Otherwise, the model specifications may need to be revised by reducing the number of interactions.

While smoothing curves can ease interpretation for sufficiently long time series, the method is not intuitively understood by those not trained in the field of statistics. Furthermore, there is no straightforward way to estimate confidence intervals for the predictions made with the GAM model. The confidence intervals would need to account for uncertainty arising from the estimation of the smoothing parameter and for survey design. There are existing approaches to capturing uncertainty in the estimation of the smoothing parameter. These include the utilization of Bayesian intervals (60, 61) and simultaneous calculation of confidence intervals (62). However, an approach also incorporating uncertainty from the survey design does not exist to the authors’ knowledge. As the motivation for calculating smoothing curves was to support a better interpretation of the moving averages, we did not aim to resolve this issue. Instead, point-wise confidence intervals are provided for each of the three-month predictions.

The interpretation of time trends should be derived from both the three-month moving estimates and the smoothed curve using their respective strengths. For example, in Figure 5, the moving averages allow for a better visual detection of change points: While the curve shows a steady increase between July/August 2022 and end of 2022, the moving averages show that the development was actually more consistent until the end of the year and only increased then. The strength of the smoothing splines is the visual identification of trends when fluctuation is high (Figure 6).

The predictions with a standard population ensure that the estimates can be directly standardized between the subgroups but also between the different time periods. Figure 6 shows the standardized and unstandardized stratified results. With our data, the differences are minimal; however, this might change with different data sources. While the unstandardized time series can be used to examine the development in real population subgroups, the standardized results can be used to better identify vulnerable groups. Both outputs provide important information for the planning of interventions for mental health promotion and prevention.

## 4. Discussion

The COVID-19 pandemic brought about a need for high-frequency monitoring of developments in the mental health of populations. Against this background, we expanded our range of strategies for a Mental Health Surveillance in Germany to include monthly reporting on a small set of indicators aiming to provide policymakers and practitioners in the health care system with high-frequency updates on developments in population mental health in order to enable swift public health action. We defined six criteria for the prototype presented in this paper and developed an infrastructure which creates new estimates as soon as new data becomes available (criterion 1). At present, data processing has to be initiated manually. However, the checks for new data could easily be automated using a task manager. With the programmed ggplot functions, graphical output can also be created automatically. The estimates are in a format and structure that can easily be fed into further applications such as databases and dashboards (criterion 7). The weighting procedure developed by the EDC allows for representative estimates for the adult population in Germany (criterion 2). As an additional safeguard against deviations between the sample distribution and the population distribution, the estimation procedure includes predictions based on the latest available estimates of the German population. This technique also allows for standardization between subgroups and over time (criteria 3 and 4). To ease graphical interpretation, the prototype produces two types of output. First, it calculates moving three-month predictions which can be considered as moving averages. Moving averages are intuitively understood and provide some smoothing of the data, but are still sensitive to short-term fluctuations. To facilitate the differentiation between signal and noise and make trends more visible, a smoothing curve is also calculated (criterion 6). The techniques developed also address our problem of few observations per month and temporal gaps in the data while optimizing temporal resolution (criterion 5). Our prototype therefore fulfills the criteria specified and is in use within the MHS (56).

The adaption of this prototype to other surveys is straightforward if the survey design remains the same over time and data has been adequately prepared for further processing. Basic data cleaning is not included in the data pipeline because the prototype was built for data from telephone surveys conducted by the EDC of the Robert Koch Institute, and initial processing of the raw data is handled within this established framework. However, the prototype does include individual scripts to homogenize the different surveys used (GEDA19/20, COVIMO, GEDA 2021, GEDA 2022). Thus, new scripts for other data sources can easily be added within the existing framework.

However, caution is warranted in the application of the prototype to multiple data sources. Minor changes in the main scripts suffice to adapt the analyses to changes in sampling designs (for example, multiple-stage sampling). However, the prototype cannot be as easily modified for other changes in survey design or data collection that might influence sample composition over time. In other words, the potential impacts of changes in data source on results should be carefully considered in applying this prototype.

Because the prototype performs prediction and standardization using a predefined marginal distribution of the German population, it includes a procedure that can correct for biases due to different study participation probabilities in certain population groups. As a consequence it can be used on unweighted data. The marginal distributions of the population used can easily be replaced by the latest or most appropriate available versions of public statistics. However, so far, the models employed for prediction are only adjusted for age group, sex, and level of education. If data is, for example, oversampled for people with a history of migration, the implemented procedure will not correct for this. Furthermore, it should be noted that a modification of the marginal distribution used for prediction and standardization will change results for the entire time series. While this ensures standardization between years, it should be noted that estimates are representative of the population only for the respective microcensus year. To achieve population representative estimates for all years in the time series, the prototype can be modified to standardize separately for each year using the respective microcensus datasets and not for the whole time series.

In the further development of the prototype, greater flexibility for the covariates should be an aim. For example, age or education groups have to be customizable for the expansion of this type of mental health surveillance for children and adolescents. This is also relevant for applications of the prototype to other health topics which might require stratification by different levels or different characteristics. A few adjustments were already made in the application of the prototype to the assessment of developments in child and adolescent mental health using data from the study “Kindergesundheit in Deutschland aktuell” [German Children’s Health Update, (63)].

We encountered several difficulties owing to data transformation between SAS, STATA, and CSV throughout the calculation process. The implementation of STATA’s margins command, including our specific use case, in R would reduce the variety of different data types to be processed This translation would need to cover the following three steps: first, the integration of probability weights for model estimation and for prediction; second, the calculation of confidence intervals for the means of the predictions; and third, the calculation the logit transformed confidence interval. A promising solution could be the R package marginal effects (64), which could potentially be integrated in the future.

As a smoothing technique to facilitate visual interpretation of the time series, we decided to use a general additive model with a smoothing thin plate spline. It produces curves that rapidly adapt to new trends emerging from new data, which also means that predictions for time periods further in the past do not change abruptly. Furthermore, the smoothing parameter of the spline can be estimated, resulting in a curve which neither over-nor underfits the data, whereas the cubic regression spline seems to excessively smooth and thus underfits. However, neither type of spline offers an advantage over the three-month predictions for short time series: they tend to be too sensitive to short-term changes in shorter time series to provide a sufficiently smoothed fit. We aim to further monitor the development of smoothing curves in these time series and define criteria for the length of time series at which the weekly smoothed predictions can be reported alongside the moving three-months average predictions.

A caveat in combining two representations of time series – a thin plate smoothing spline and three-month average estimates – is that there is more and potentially conflicting information to take into consideration in the process of visual interpretation. These two representations can come apart because the smoothing spline is estimated independently of average predictions for the three-month windows and estimated on weekly data in order to use all available temporal information and to achieve a curve without edges. This serves as a robustness check against overinterpretation of (random) changes in the three-month estimates. While the spline is sometimes inferior to the three-month moving averages when the goal is to visually detect change points, it is superior in separating signal from noise when there are large fluctuations in the estimated moving averages.

## 5. Conclusion

The high-frequency surveillance of health indicators using survey data can help enable rapid and flexible responses to ongoing changes in population health, particularly in times of crises. However, using survey data to assess health developments at frequent intervals poses unique data processing and analysis challenges. The prototype presented here was designed to overcome these challenges and to provide automatic monthly updates on multiple indicators on the basis of relatively small monthly samples. It will continue to serve as a basis for high-frequency surveillance of mental health within the MHS at the Robert Koch Institute (see 8) and may potentially be applied to high-frequency surveillance in other areas of health in the future. The prototype’s output is highly suitable for publication and regular updates on a dashboard, which is the next aim within the MHS. The precondition, however, is ongoing continuous data collection. While there are numerous possibilities for the further development of the prototype, some of which have been addressed above, the described approach as it stands may be of use to other researchers in public health implementing a similar type of surveillance.

## Data availability statement

The datasets presented in this article are not readily available because the population-based data from the German health monitoring program that were used for the development of the prototype presented here are available from the Robert Koch Institute (RKI) but restrictions apply to the availability of these data. The data sets cannot be made publicly available because informed consent from study participants did not cover public deposition of data. However, a minimal data set is archived in the Health Monitoring Research Data Centre at the RKI and can be accessed by all interested researchers. On-site access to the data set is possible at the Secure Data Centre of the RKI’s Health Monitoring Research Data Centre. Requests to access the datasets should be directed to fdz@rki.de.

## Ethics statement

The studies involving human participants were reviewed and approved by GEDA and COVIMO are subject to strict compliance with the data protection provisions set out in the EU General Data Protection Regulation (GDPR) and the Federal Data Protection Act (BDSG). Participation in the study was voluntary. The participants were informed about the aims and contents of the study and about data protection. Informed consent was obtained verbally. In the case of GEDA 2019/2020, the Ethics Committee of the Charité-Universitätsmedizin Berlin assessed the ethics of the study and approved the implementation of the study (application number EA2/070/19). Written informed consent for participation was not required for this study in accordance with the national legislation and the institutional requirements.

## Author contributions

SD and SJ developed the analysis methods in the paper. SJ mainly scripted the automatic pipeline while SD was in charge of the weighting procedure described in the paper. SD drafted the sections 3.2.1 and 3.2.2. SJ wrote the sections 2, 3.1, 3.2, 3.2.3, 3.2.4 and the Introduction. SD and SJ wrote section 3.2.5 together. Sections 1, 4 and 5 were written by LW, SD, and SJ. LW also edited and commented on all sections extensively. EM also commented on all sections and was in charge of the project. All authors contributed to the article and approved the submitted version.

## Funding

The “MHS – Set-up of a National Mental Health Surveillance at Robert Koch Institute” project has been funded by the Federal Ministry of Health (Grant Number: Chapter 1504 Title 54401). The studies GEDA 2019/20, GEDA 2021, COVIMO an GEDA 2022 were funded by the Robert Koch Institute and the German Federal Health Ministry.

## Conflict of interest

The authors declare that the research was conducted in the absence of any commercial or financial relationships that could be construed as a potential conflict of interest.

## Publisher’s note

All claims expressed in this article are solely those of the authors and do not necessarily represent those of their affiliated organizations, or those of the publisher, the editors and the reviewers. Any product that may be evaluated in this article, or claim that may be made by its manufacturer, is not guaranteed or endorsed by the publisher.

## Footnotes

^1^ *SAS and all other SAS Institute Inc. product or service names are registered trademarks or trademarks of SAS Institute Inc. in the USA and other countries. ® indicates USA registration.*

## Abbreviations

COVIMO: COVID-19 Impfquoten Monitoring (COVID-19 vaccination rate monitoring)
GAM: General Additive Model
GEDA: Gesundheit in Deutschland aktuell (German Health Update)
MHS: Mental Health Surveillance
